# Nurses’ self-improvement hand-hygiene compliance in a hospital ward: combining indoor location with gamification data presentation

**DOI:** 10.1186/2047-2994-4-S1-I11

**Published:** 2015-06-16

**Authors:** LV Lapao, R Marques, J Gregorio, M Mira-da-Silva

**Affiliations:** 1 International Public Health and Biostatistics, Instituto de Higiene e Medicina Tropical - Universidade Nova de Lisboa, Portugal; 2 Informatics Engineering Dept., Instituto Superior Técnico, Lisbon, Portugal

## Introduction

Healthcare acquired infections can be prevented by means of hand hygiene (HH) compliance. Nonetheless, leading busy healthcare workers to comply with HH remains puzzling. Recognized hurdles are lack of time, forgetfulness, wrong technique, lack of motivation and awereness about compliance.

## Objectives

This study aims at exploring the use of gamification to promote nurses’ HH compliance self-awareness and action. Real-time data collected from an indoor location systems will provide feed-back information to nurses working in a ward.

## Methods

A design science approach is used to design and test a solution [1]. Gamification was selected as the solution (Osyrish) to the compliance problem to engage and motivate people to achieve specific goals [2]. An innovative indoor system, based on Beacons (iBeacon™), is used to collect data on nurses’ position (and time) to enable both the detection of HH moments and its validation. Each nurse carry a device running an application that use the received signals to detect its proximity to the beacons, being able to know to which one it is closest to, thus knowing its relative position in the room. After this, data is collected to display, in anonymous way, nurses’ compliance in real-time. Changes in behavior were measured.

## Results

The compliance of HH in the ward was studied before and after the intervention. The system was installed and tested with significant precision. 35 Beacons were placed in the ward (in the room’s doors, in each alcohol-based hand rub container, in each sink and in each side of the bed). Even though times aren’t totally accurate, we are able to detect nurses’ movements using proximity and quantify compliance. Participant nurses approved the measure as an opportunity to improve their performance.

## Conclusion

The impact of gamification on HH compliance is still under evaluation. So far the results show significant improvements in nurses’ awareness. The nurses participated from the beginning enabled a higher sense of ownership in the process, recognized as a performance enabler.

## Disclosure of interest

None declared.
